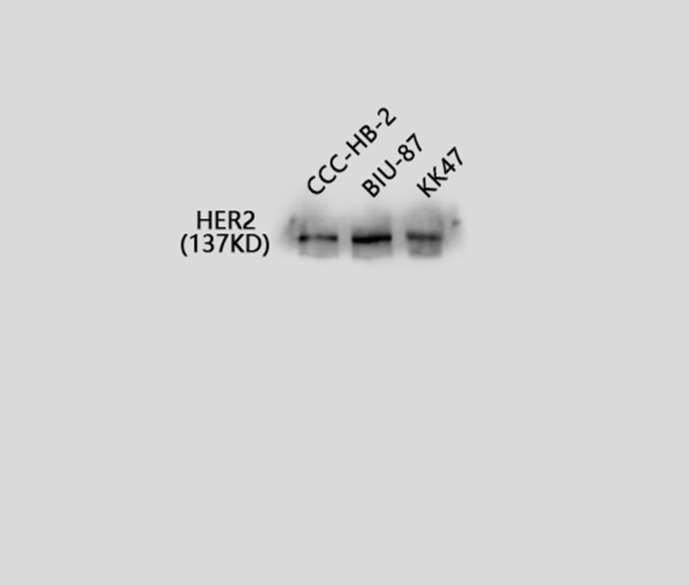# Association between TOP2A, RRM1, HER2, ERCC1 expression and response to chemotherapy in patients with non-muscle invasive bladder cancer

**DOI:** 10.1016/j.heliyon.2022.e09643

**Published:** 2022-06-08

**Authors:** Zhifei Liu, Liyong Xing, Yanfeng Zhu, Peng Shi, Gang Deng

**Affiliations:** Department of Urology, Tangshan People's Hospital, Hebei 063001, China

**Keywords:** Bladder cancer, TOP2A, RRM1, ERCC1, Chemotherapy

## Abstract

**Purpose:**

This study aimed to detect the expression levels of topoisomerase IIα (TOP2A), ribonucleotide reductase catalytic subunit M1 (RRM1),c-erbB-2 (HER2) and excision repair cross complementing group 1 (ERCC1) in non-muscular invasive bladder cancer (NMIBC) and explore the correlation between the expression of these genes and NMBIC sensitivity to pirarubicin or gemcitabine treatment.

**Materials and methods:**

NMIBC patient tissues and the bladder cancer cell lines BIU-87 and KK47 were selected for the exploration of drug sensitivity in vitro. Immunohistochemistry was used to examine protein expression in tissues. Reverse transcription-polymerase chain reaction (RT-qPCR) and a Western blot assay were used to detect the mRNA and protein levels in cells. The cell IC50 value was evaluated by an MTT assay. Flow cytometry was used to sort the cell subpopulations.

**Results:**

In the pirarubicin-treated group, the patients with high TOP2A expression experienced lower recurrence rates than those with low TOP2A expression, whereas TOP2A and HER2 co-expression resulted in higher recurrence rates. The patients with low RRM1 expression, especially those with low ERCC1 expression, experienced lower recurrence rates than the patients with high RRM1 expression in the gemcitabine-treated group. Tumour cells with high TOP2A expression were highly sensitive to pirarubicin, and TOP2A^+^ HER2^-^ cells were more sensitive to pirarubicin than TOP2A^+^ HER2^+^ cells. Cells with low RRM1 expression levels were sensitive to gemcitabine, and RRM1^−^ERCC1^-^ cells were more sensitive to gemcitabine than RRM1^−^ERCC1^+^ cells.

**Conclusion:**

High TOP2A expression or low RRM1 expression could predict the sensitivity of NMIBC to pirarubicin or gemcitabine treatment. HER2 and ERCC1 expression may affect the effect of TOP2A and RRM1, thus affecting the efficacy of chemotherapeutic drugs.

## 1 Introduction

Bladder cancer is a common malignant tumour of the urinary tract in men, and in most cases, tumour cells do not invade the muscle. These tumours are called non-muscle invasive bladder cancer (NMIBC). Although NMIBCs have high recurrence rate, intravesical instillation therapy for NMIBC after transurethral resection can efficiently reduce the recurrence rate from 70% to 20% [1]. Effective intravesical chemotherapy is critical for the treatment of bladder cancer. Anthracyclines (e.g., pirarubicin) and pyrimidines (e.g. gemcitabine) are frequently used in intravesical chemotherapy for NMIBC after transurethral resection [2, 3]. Although these agents are not precise molecular-targeted drugs, some tumours with the overexpression of specific genes are highly sensitive to these treatments. TOP2 is one isoform of the Topoisomerase II (TOP2A) which is a nuclear protein required for DNA replication and cell division. TOP2A is a potentially important chemotherapy target, and several of anticancer drugs, including anthracyclines, act on this gene [4, 5, 6]. In patients with medulloblastoma and breast cancer, tumours with high TOP2A expression levels are sensitive to anthracyclines and these drugs show good therapeutic effects. Decreased TOP2A activity or expression may lead to tumour resistance to TOP2A inhibitors [7, 8]. TOP2A was significantly up-regulated in high-grade and advanced stage bladder urothelial carcinoma (BLCA) samples than in normal epithelial tissue. High TOP2A expression patients had poorer cancer-specific, progression-free and recurrence-free survival [9]. Ribonucleotide reductase large subunit M1 (RRM1) is a rate-limiting enzyme that catalyzes the conversion of ribonucleotide to dNTPs, contributes to DNA repair, and cell growth because of the role in de novo DNA synthesis during cell replication [10]. RRM1 is an important molecular target of gemcitabine because it can control the specificity of a substrate and the activity of an enzyme. Patients with low RRM1 expression had a relatively long survival times after chemotherapy [11, 12]. In NMIBC patients treated with intravesical gemcitabine monotherapy, low RRM1 expression patients had longer progression-free survival and lower 1-year/2-year relapse rates, although there is a need to further verify the results [13]. Pirarubicin and gemcitabine are the most commonly used chemotherapeutic drugs. However, minimal research has been carried out to investigate the correlation between TOP2A and RRM1 expression and the pharmacodynamics of the aforementioned drugs in NMIBC. Identifying markers for the prediction of treatment sensitivity in patients receiving pirarubicin or gemcitabine chemotherapy is of great value.

Human epidermal growth factor receptor 2 (HER-2) is a transmembrane receptor, it is involved in malignant tumors progression including BLCA [14]. It is the main pathogenic proto-oncogene of breast cancer, and plays an important role in its occurrence and development. In many tumours, HER-2 and TOP2A are co amplified and expressed, indicating that the activation of multiple genes determines the biochemical activity and clinical characteristics of tumour cells [15]. Many studies have shown that the configuration of 17q21 amplicon in cancer cells may influence the effect of HER-2 on TOP2A inhibitors, thereby affecting the efficacy of these chemotherapeutic drugs [16]. Further studies are needed to confirm whether a relationship exists between HER-2 and TOP2A in bladder cancer. Excision repair cross complement group 1 (ERCC1) identifies and resects the damaged DNA strand that may be representative for the crucial DNA damage repair ability of the cell. It is an important factor involved in nucleotide excision repair [10]. Reynolds et al. found that a significant correlation exists between the expression levels of RRM1 and ERCC1 protein and the response rate to chemotherapy, and the response rate was low in patients with a high expression of these proteins [17, 18]. The role of pirarubicin and gemcitabine in postoperative bladder infusion chemotherapy for bladder cancer patients needs further investigation.

In this study, we explored the correlation between TOP2A, RRM1, HER2 and ERCC1 expression levels and the sensitivity of NMIBC to pirarubicin or gemcitabine treatment. Effective chemotherapeutic agents could improve the treatment outcomes of NMIBC patients.

## 2 Materials and Methods

### 2.1 Patients

In total, 85 Ta, Tis and T1 tumour (TNM) urothelial carcinoma samples of low-grade papillary urothelial carcinoma, and high-grade papillary urothelial carcinoma were collected from patients who underwent transurethral resection of bladder tumour (TURBT) from April 2012 to March 2017 at Tangshan People's Hospital. There were 63 males and 22 females, aged 33–88 years, with an average age of 67.87 years. Two pathologists made pathological diagnosis independently; The overall survival rate of the patients was estimated in this study. Patents follow-up time range from 6 months to 108 months, median time was 53.81 months. Additionally, after TURBT, 45 patients underwent IVT (intravesical therapy) with pirarubicin, whereas 40 patients underwent IVT of gemcitabine. The recurrence rates of the tumours were calculated within one year. All protocols were conducted in accordance with the ethical guidelines of the 1975 Helsinki Declaration, and the use of tissue samples in this study was approved by the Ethical Committee of Tangshan People's Hospital (No. 20171274). Written informed consent was obtained from each participant according to institutional guidelines.

### 2.2 Immunohistochemistry

Eighty-five paraffin-embedded NMIBC tissue sections were cut into 5 μm thick sections. Then, the sections were dewaxed in xylene and hydrated through a serial alcohol gradient. Antigens were removed by microwaving in citrate buffer (pH 6.0) for 15 min. The sections were placed in the endogenous peroxidase inhibitor 3% H_2_O_2_ for 30 min to eliminate endogenous peroxidase activity. Then, the sections were incubated with the primary antibodies at 4 °C in a refrigerator overnight. The sections were washed with PBS buffer 3 times (3 min per wash), and the sections were incubated with a secondary antibody at room temperature for 60 min. Then, 3, 30-diaminobenzidine (DAB) developer was added for development. Three high-power fields (40×) were randomly selected for the cell counts and the staining intensity was evaluated and scored. Immunohistochemical results were interpreted by evaluating the staining intensity and frequency of positive tumour cells (mean score). The final score was determined by multiplying the staining intensity score and the frequency scores of the positive area. The intensity scores were defined as 0 to 3 (negative, weak; moderate; and strong). The frequency of positive cells was defined as 0 to 4 (less than 5%; 5%–25%; 26%–50%; 51%–75%; and 4, greater than 75%). Scores ≤7 were considered negative or low expression, and scores of ≥8 were considered positive or high expression.

### 2.3 Antibodies and drugs

Anti-TOP2A antibody (1:400; ab52934, Abcam, Cambridge, MA, USA), anti-RRM1 antibody (1:200; ab137114, Abcam), anti-ErbB2/HER2 antibody (1:400; ab2645411, Abcam) and anti-ERCC1 antibody (1:400; ab2356, Abcam) were used in this study. Gemcitabine (catalogue no. HY-B0003, MedChemExpress, USA) and pirarubicin (catalogue no. HY-13725A, MedChemExpress, USA) were used.

### 2.4 Cell lines

The NMIBC cell lines BIU-87 and KK47, were cultured in RPMI-1640 medium (Key Gene Biotech, Nanjing, CN) with 10% foetal bovine serum (Biological Industries, Kibbutz Beit-Haemek, ISRAEL), supplemented with and 1% streptomycin sulfate and penicillin sodium at 37 °C and 5% CO_2_. These two cell lines were purchased from the National Infrastructure of Cell Line Resource (Chinese Academy of Medical Sciences).

### 2.5 Plasmid transfection

TOP2A was knocked down in BIU-87 cells by transfection with a TOP2A shRNA plasmid (catalogue no. HSH018172-LVRH1P, GeneCopoeia, USA), and RRM1 was overexpressed in KK47 cells by transfection with a RRM1 cDNA plasmid (catalogue no. EX-C0244-Lv105-10, GeneCopoeia, USA). The Lenti-Pac™ HIV expression kit (catalogue no. HPK-LvTR-20, GeneCopoeia. USA) was used for the plasmid transfection according to the manufacturer's directions. Transfection efficiency was evaluated by quantitative reverse transcription-polymerase chain reaction (RT-qPCR) and a Western blot analysis.

### 2.6 RNA isolation and RT-qPCR

The total RNA was extracted using TRIzol reagent (Tiangen Biotech, Beijing, China), and RT-qPCR was performed as previously described [19]. The primers for TOP2A (catalogue: HQP018172), RRM1 (catalogue: HQP054684) and GAPDH (catalogue: HQP006940) internal controls were constructed by GeneCopoeia. PCR was conducted by an ABI 7500 Real-Time PCR System. GAPDH was used as an internal reference. Genes expression were calculated by the 2^−ΔΔCt^ method as follows: △△Ct=(average Ct value of the target gene in the experimental group-average Ct value of the GAPDH in the experimental group)-(average Ct value of the target gene in the control group-average Ct value of the GAPDH in the control group). A 2^−ΔΔCt^ value > 1 was considered high expression, and a value < 1 was considered low expression.

### 2.7 Western blot analysis

Protein sample preparation: One millilitre of RIPA lysate with 10 μl PMSF were used for the cell lysis, and an appropriate amount of lysate was added to the cells and the cells were incubated on ice for 30 min. The proteins were separated by electrophoresis based on their molecular weight using SDS–PAGE, and transferred from the gel to a PVDF membrane. The nonspecific binding site was blocked by immersing the membrane in 5% non-fat milk in TBS solution for 1 h at RT. The membrane was incubated overnight at 4 °C, washed with TBS buffer and incubated with a secondary antibody for 1 h at RT. GAPDH was taken as internal reference. ImageJ analysis software was used to quantify the greyscale of each band in the Western blot images. The grey value was standardized by GAPDH, and the protein expression fold difference = protein grey value/GAPDH grey value. A fold difference>1 was considered high expression, and a value < 1 was considered low expression.

### 2.8 MTT assay

The cell survival ratio was determined by an MTT assay. The cells were seeded into 96-well plates, cultured for 24 h and then incubated with fresh medium supplemented with various concentrations of pirarubicin (0, 0.5, 1, 2, 4 and 8 μg/ml) or gemcitabine (0, 1, 3, 5, 7 and 10 μg/ml). After 48 h, 20 μl of MTT (5 mg/ml, Sigma–Aldrich) were added for 4 h. The supernatant was then removed and 200 μl of DMSO were added to dissolve the formazan. The viable cells were detected by a 96-well micro plate reader (Bio-Rad, Hercules, CA, USA) at a wavelength of 490 nm. The survival ratio was calculated as follows: survival ratio (%) = (OD of the drug treated group)/(OD of the control group). The half maximal inhibitory concentration (IC50) was defined as the concentration of the drug that resulted in 50% inhibition of cell growth and was calculated using a linear regression analysis. All experiments were repeated three times.

### 2.9 Flow cytometry and cell sorting

BIU-87 cells were collected and incubated with antibodies against TOP2A and HER2 or RRM1 and ERCC1 (1:100 dilutions) at 4 °C for 45 min. A FITC-conjugated rabbit anti-mouse IgG antibody and PE-conjugated goat anti-rabbit antibody were used as secondary antibodies, and the cells were incubated for 45 min at room temperature in the dark. The cells were washed three times and re-suspended in 500 μl of PBS. The samples were analysed and sorted by fluorescence activated cell sorter (FACS) Calibur flow cytometer (BD Bioscience) with Cell Quest software (BD Biosciences).

### 2.10 Statistical analysis

The data analysis was performed with SPSS 17.0 software. To compare the correlations between TOP2A, HER2, RRM1 or ERCC1 expression and the tumour recurrence rates, measurement data were expressed by mean ± standard deviation. Student's t-test is used to compare gene expression between two groups. We applied a chi-square test to assess the categorical variables and adjusted them by Fisher's exact test and Yates' correction. The overall survival (OS) rate was calculated by the Kaplan-Meier method. The differences in the survival curves were evaluated by a log-rank test. The P-values were two-sided, and statistical significance was determined at the 0.05 level.

## 3 Results

Expression of TOP2A and RRM1 in human NMIBC tissues and its significance in cancer recurrence after treatment with pirarubicin or gemcitabine

**Figure 1:**
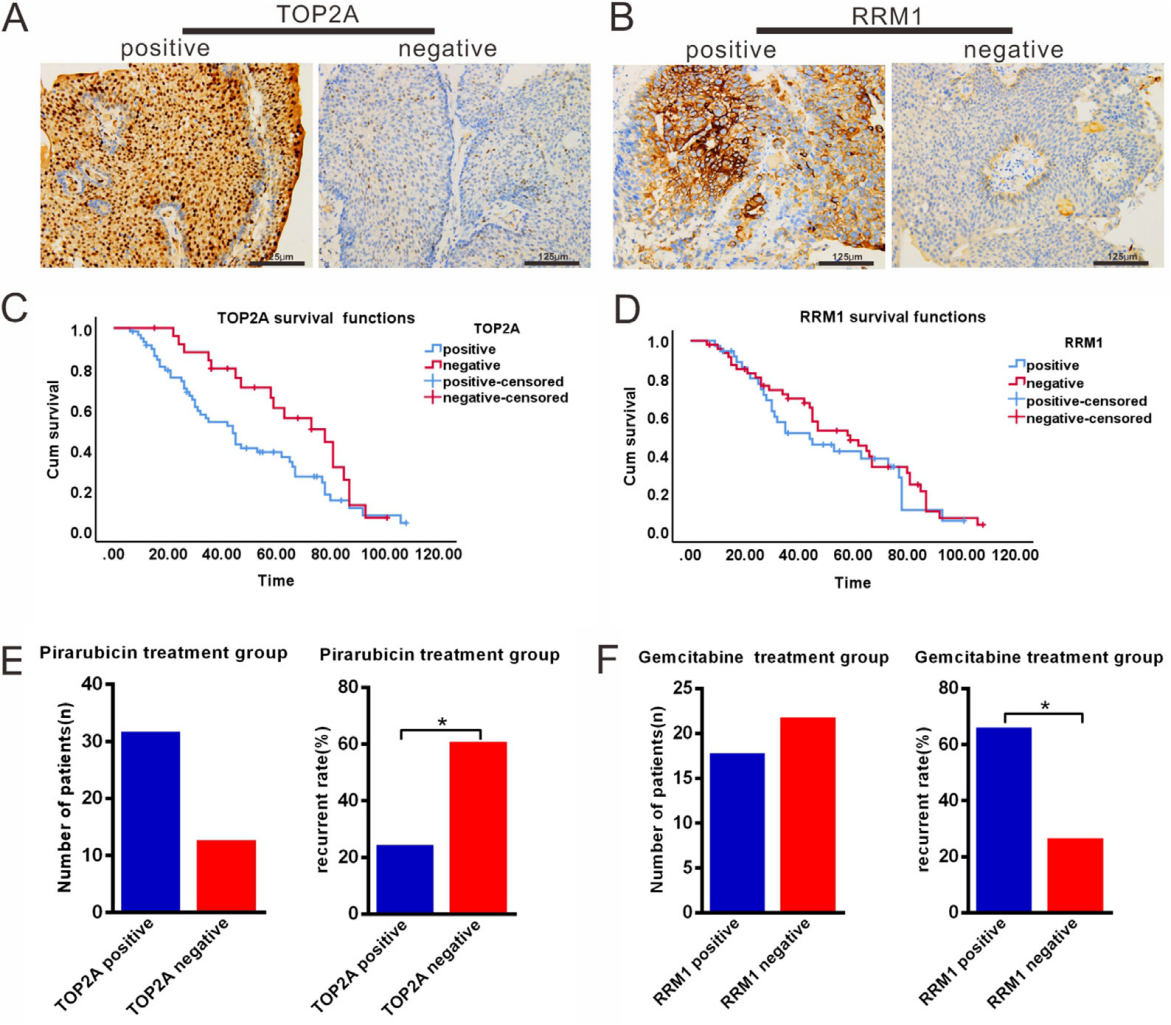
Expression levels of TOP2A and RRM1 in human NMIBC tissues and their significance in cancer recurrence. A and B, Immunohistochemistry show positive and negative expression of TOP2A and RRM1 in NMIBC tissues. C, The mean survival time of the TOP2A-positive group was significantly shorter than that of the TOP2A-negative expression group (p = 0.040). D, No significant difference was observed in the overall survival rates between the RRM1-positive and RRM1-negative expression groups (p > 0.05). E, In the pirarubicin-treatment group, 32 and 13 patients had positively and negatively expressed TOP2A, respectively. Tumours with high TOP2A expression showed a lower recurrence rate than those with low TOP2A expression after one year of pirarubicin treatment (p = 0.037). F, In the gemcitabine-treatment group, 18 and 22 patients had positive and negative RRM1 expression, respectively. Tumours with low expression of RRM1 had lower recurrence rate than tumours with high expression of RRM1 after treatment with gemcitabine within one year (p = 0.024).

To investigate the relationship between TOP2A and RRM1 expression and cancer recurrence after treatment with pirarubicin or gemcitabine, we collected 85 NMIBC patient tissues obtained during TURBT. The tumours of fifty-nine patients positively expressed TOP2A, 13 tumours negatively expressed TOP2A (Figure 1A), and 37 tumours positively expressed RRM1 (Figure 1B). The association between TOP2A and RRM1 expression and the patient overall survival rate was evaluated. The mean survival time of the TOP2A-positive group was 48.493 ± 4.097 months, which was significantly shorter than that of the TOP2A-negative expression group (66.413 ± 4.993) (p = 0.040; Figure 1C). However, no significant difference was observed in the overall survival rates between the RRM1-positive and RRM1-negative expression groups (p = 0.431, Figure 1D). Due to the small sample size in the sub-group, more cases need to be collected for further research.

To investigate the relationship between TOP2A and RRM1 expression and cancer recurrence after treatment with pirarubicin or gemcitabine, in total, among 45 patients who underwent intravesical chemotherapy with pirarubicin, 32 had tumours that positively expressed TOP2A and 13 had tumours that negatively expressed TOP2A (Figure 1E). In the pirarubicin-treated group, the recurrence rate in the patients with positive TOP2A expression was 25%, whereas that in patients with negative TOP2A expression was 61.5% within one year (p ≤ 0.05, Figure 1E, Table 1). Forty patients underwent intravesical chemotherapy with gemcitabine; of these patients, 18 had tumours that positively expressed RRM1, and 22 had tumours that negatively expressed RRM1 (Figure 1F, Table 2). Among the 40 patients treated with gemcitabine, the recurrence rate of tumours with RRM1 overexpression was 66.7%, whereas the recurrence rate of tumours with low RRM1 expression was 27.3% (p ≤ 0.05, Figure 1F). These results indicated that pirarubicin had a considerable effect on tumours with high TOP2A expression. Gemcitabine was effective in tumours with low RRM1 expression.

**Table 1:** Relationship between TOP2A expression and recurrence rate after treatment with pirarubicin

Recurrent state	TOP2A		*p*
	High expression (%)	Low expression (%)	
Recurrence	8 (25%)	8 (61.5%)	0.037∗
NO recurrence	24 (75%)	5 (38.5%)	

∗*p* ≤ 0.05.

**Table 2:** Relationship between RRM1 expression and recurrence rate after treatment with gemcitabine.

Recurrent state	RRM1		*P*
	High expression (%)	Low expression (%)	
Recurrence	12 (66.7%)	6 (27.3%)	0.024∗
NO recurrence	6 (33.3%)	16 (82.7%)	

∗*p* ≤ 0.05.

Sensitivity of cell lines with different expression levels of TOP2A and RRM1 to different drugs

**Figure 2:**
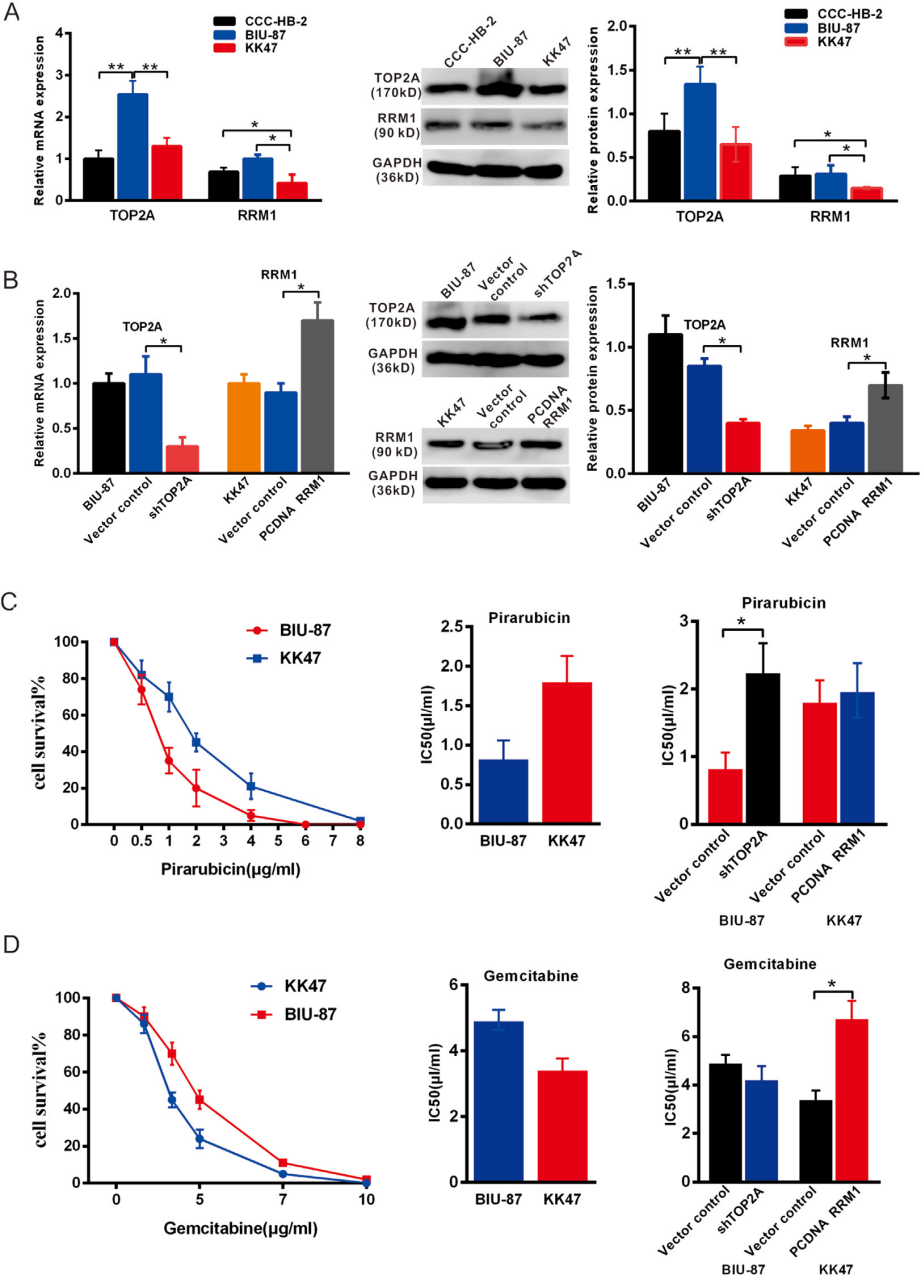
mRNA and protein levels of TOP2A and RRM1 in bladder cancer cell line cells. A, RT-PCR and Western blot analysis showed that the mRNA and protein levels of TOP2Ain the BIU-87 cell line were higher than those in the CCC-HB-2 and KK47 cell line (p < 0.01), whereas the mRNA and protein levels of RRM1were lower in the KK47 cells (p < 0.05) (The original blots images were provided in supplement figure1A-TOP2A, supplement figure1A-RRM1 and supplement figure1A-GAPDH). B, The mRNA and protein levels of TOP2A in BIU-87 cells (The original blots images were provided in supplement figure1B-TOP2A, supplement figure1B-GAPDH-TOP2A, supplement figure1B-RRM1 and supplement figure1B-GAPDH-RRM1) were significantly decreased by the transfection with TOP2A shRNA, and RRM1 was increased in KK47 cells by the transfection with the PCDNA3 RRM1 plasmid. C, Survival curves of BIU-87 and KK47 cells treated with different concentrations of pirarubicin (0, 0.5, 1, 2, 4 and 8 μg/ml) for 48 h. The IC50 values of pirarubicin in BIU-87 and KK47 cells were 0.84 ± 0.22 μg/ml and 1.82 ± 0.31 μg/ml, respectively. The IC50 values of pirarubicin in the shTOP2A group were significantly higher than those in the vector control group of BIU-87 cells (p < 0.05). D, Survival curves of BIU-87 and KK47 cells treated with different concentrations of gemcitabine (0, 1, 3, 5, 7 and 10 μg/ml) for 48 h the IC50 values of gemcitabine in BIU-87 and KK47 cells were 4.94 ± 0.31 μg/ml and 3.45 ± 0.32 μg/ml, respectively. The IC50 values of gemcitabine in the PCDNA RRM1 group were significantly higher than those in the vector control group of KK47 cells (p < 0.05). Each experiment was repeated three times.

To determine whether TOP2A and RRM1 expression plays a role in tumour drug sensitivity, we measured the mRNA and protein levels of TOP2A and RRM1 in the bladder tumour cell lines BIU-87, KK47 and human embryonic bladder tissue derived cells CCC-HB-2 by RT-qPCR and Western blot analysis. The results showed that TOP2A was more highly expressed in the BIU-87 cells than in the CCC-HB-2 and KK47 cells, while RRM1 expression was lower in KK47 cells, and its expression was found to be low in both cell lines (Figure 2A). We selected BIU-87 cells to evaluate the effect of down regulating TOP2A and KK47 cells to evaluate the effect of up regulating RRM1 to estimate the correlation between the expression of these two genes and tumour sensitivity to pirarubicin and gemcitabine (Figure 2B).

The MTT assay showed that the IC50 values of pirarubicin in the BIU-87 and KK47 cells were 0.84 ± 0.22 μg/ml and 1.82 ± 0.31 μg/ml, respectively. BIU-87 cells with high TOP2A expression were more sensitive to pirarubicin than gemcitabine, and the down regulation of TOP2A in the BIU-87 cells decreased sensitivity to pirarubicin (p ≤ 0.05, Figure 2C). The IC50 values of gemcitabine in the BIU-87 and KK47 cells were 4.94 ± 0.31 μg/ml and 3.45 ± 0.32 μg/ml, respectively, and KK47 cells with low RRM1 expression had more effective response to gemcitabine (p ≤ 0.05). The up regulation of RRM1 in the KK47 cells decreased the inhibitory capacity of gemcitabine (Figure 2D). These results further confirm that TOP2A is associated with sensitivity to pirarubicin, whereas RRM1 is correlated with sensitivity to gemcitabine.

### 3.1 The expression of HER2 and ERCC1 in NMIBC and its relationship with chemotherapy

**Figure 3:**
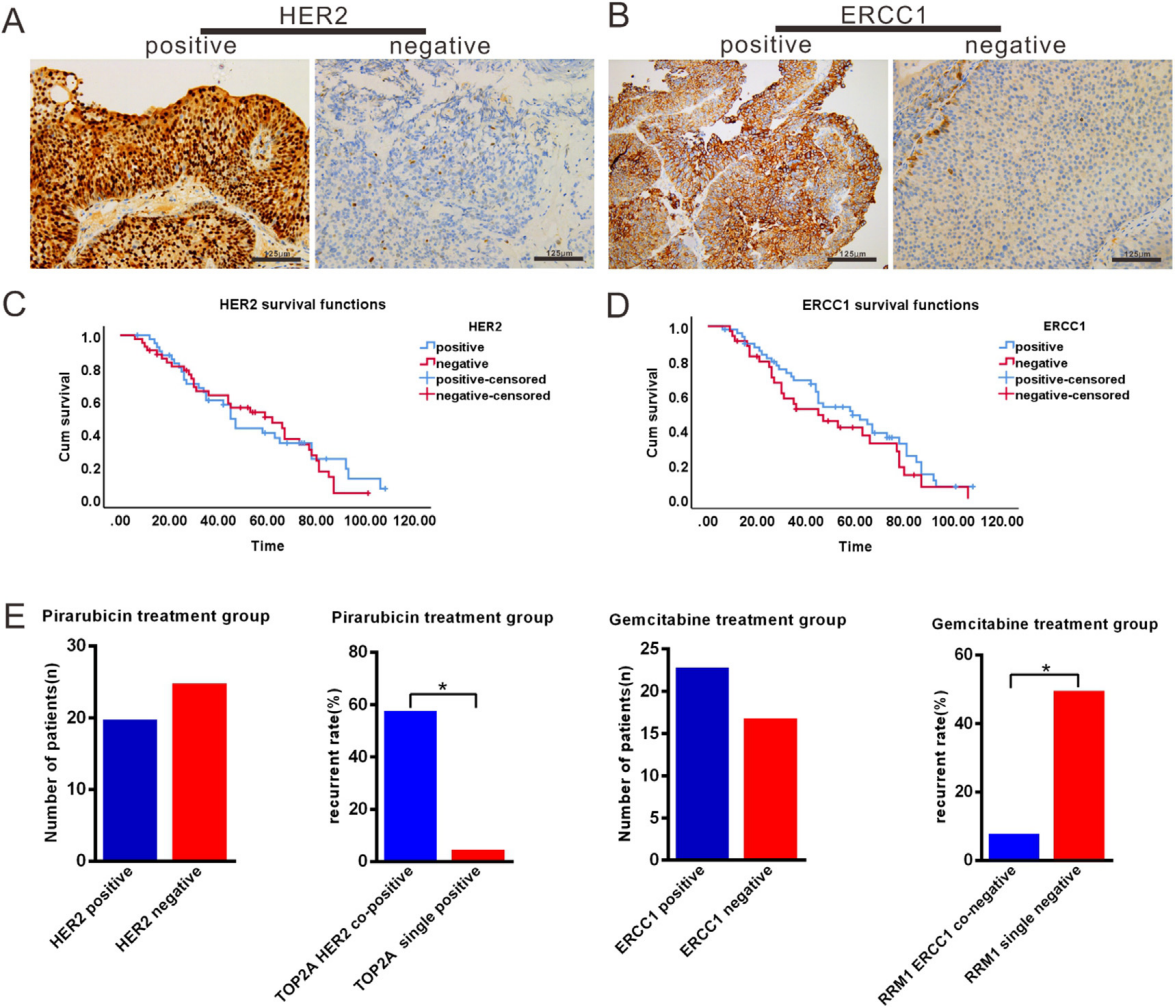
Expression levels of HER2 and ERCC1 in human NMIBC tissues and their significance in cancer recurrence. A, Immunohistochemistry shows positive and negative expression of HER2 in NMIBC tissues. B, Immunohistochemistry shows positive and negative expression of ERCC1 in NMIBC tissues. C, No significant difference was observed in the overall survival rates between the HER2-positive and HER2-negative expression groups (p > 0.05). D, No significant difference was observed in the overall survival rates between the ERCC1-positive and ERCC1-negative expression groups (p > 0.05). E, In the pirarubicin-treatment group, HER2 was positively expressed in 20 of the 45 patients. The recurrence rate in the patients with positive TOP2A and HER2 co-expression was higher than that in those with TOP2A expression alone (p = 0.002). F, In the gemcitabine-treatment group, ERCC1 was negatively expressed in 17 of the 40 cases. Patients with both the negative expression of both RRM1 and ERCC1 had lower recurrence rates than those with only a low expression of RRM1 (p = 0.023).

Researchers have suggested that the HER2 status may influence the effect of TOP2A on inhibitors on cancer cells, and a significant correlation exists between the expression of RRM1 and ERCC1 and response to chemotherapy. Therefore, the expression of HER2 and ERCC1 in NMIBC was detected. HER2 was mainly expressed in the nucleus (Figure 3A), and positive expression was observed in 41 of 85 tumour samples, while ERCC1 expression was positive in 50 tumour samples (Figure 3B). Neither HER2 nor ERCC1 expression was correlated with the overall survival rate (p > 0.05, Figure 3C, D). We further detected a correlation between the expression of HER2 and TOP2A and that of RRM1 and ERCC1 in relation to chemotherapy sensitivity. HER2 positive expression was observed in 20 of the 45 patients who underwent intravesical chemotherapy with pirarubicin (Figure 3E). The recurrence rate in the patients with positive TOP2A and HER2 co-expression (58.3%) was higher than that in the patients with TOP2A single expression (5.3%) (p ≤ 0.05, Figure 3E, Table 3). ERCC1 was mainly expressed in the cytoplasm and negatively expressed in 17 of the 40 patients who underwent intravesical chemotherapy with gemcitabine (Figure 3F). The patients negatively expressing both RRM1 and ERCC1 had a lower recurrence rate (8.3%) than those with only RRM1 negative expression (50%). (p ≤ 0.05, Figure 3F, Table4).

**Table 3:** Relationship between TOP2A and HER2 expression and recurrence rate after treatment with pirarubicin

Recurrent state	TOP2A HER2	TOP2A	*p*
	Co-expression (%)	Single high expression (%)	
Recurrence	7 (58.3%)	1 (5.3%)	0.002∗
NO recurrence	5 (41.7%)	19 (94.7%)	

∗*p* ≤ 0.05.

**Table 4:** Relationship between RRM1 and ERCC1 expression and recurrence rate after treatment with gemcitabine.

Recurrent state	RRM1 ERCC1	RRM1	*p*
	Co-low expression (%)	Single low expression (%)	
Recurrence	1 (8.3%)	5 (50%)	0.023∗
NO recurrence	11 (91.7%)	5 (50%)	

∗*p* ≤ 0.05.

### 3.2 Sensitivity of different cell subpopulations to drugs

**Figure 4:**
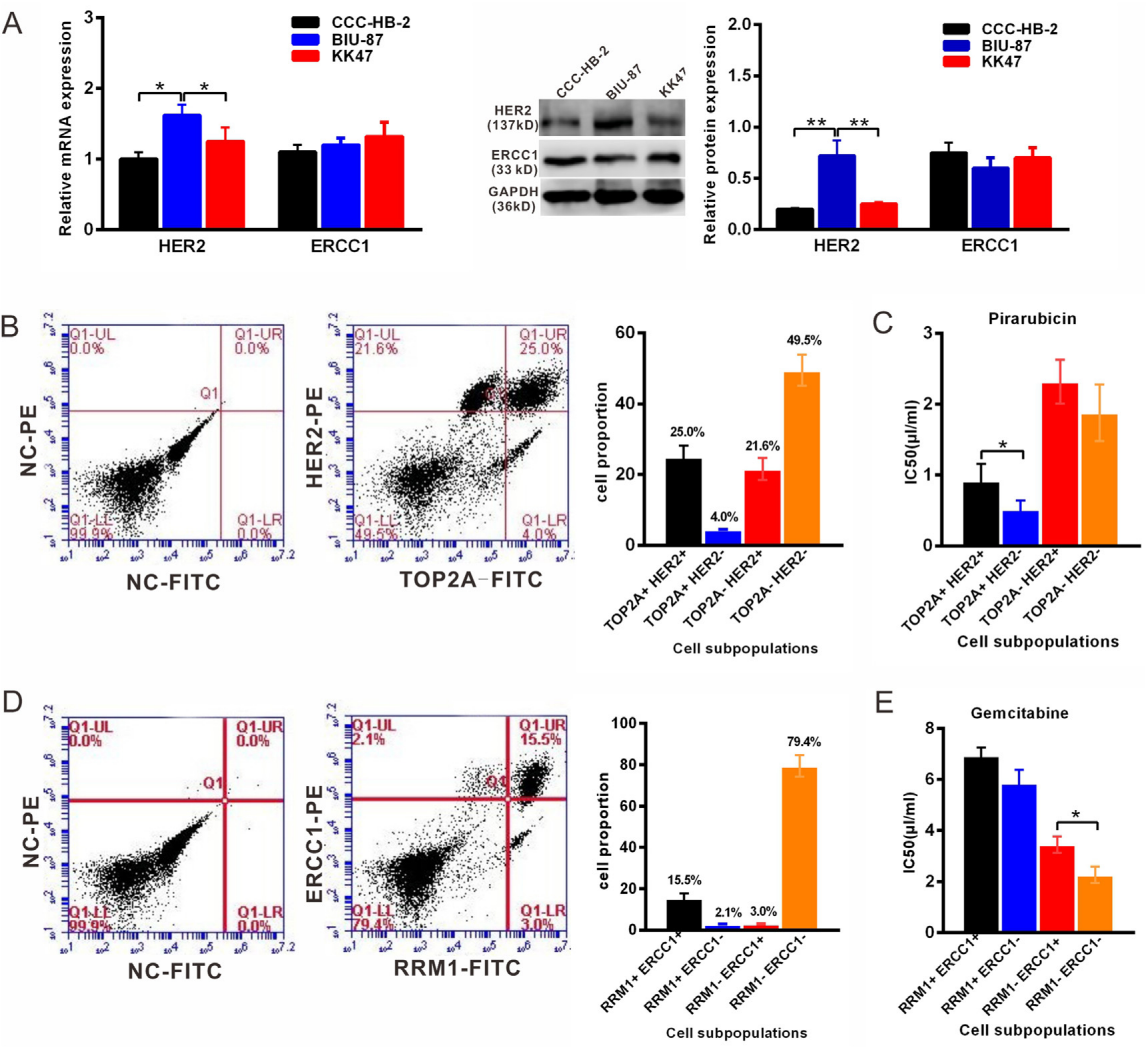
The sensitivity of different subpopulations of cells to chemotherapeutic drugs. A, RT-PCR and Western blot analysis showed that the mRNA and protein levels of HER2 in the BIU-87 cell line were higher than those in the CCC-HB-2 and KK47 cell line, whereas the mRNA and protein levels of RRM1 were no significant different in these cells (The original blots images were provided in supplement figure2A-HER2, supplement figure2A-ERCC1 and supplement figure2A-GAPDH). **B,** TOP2A^+^HER2^-^ cells, TOP2A^+^HER2^+^ cells, TOP2A^−^HER2^+^ cells and TOP2A^−^HER2^-^ cells were sorted from BIU-87 cells by FACS. **C,** Separated TOP2A^+^ HER2^-^ cells were more sensitive to pirarubicin than TOP2A^+^HER2^+^ positive cells (p < 0.05). **D,** RRM1^+^ERCC1^-^ cells, RRM1^+^ERCC1^+^ cells, RRM1^−^ERCC1^+^cells and RRM1^−^ERCC1^-^ cells were also sorted from the BIU-87 cell line. **E,** RRM1^−^ERCC1^-^ cells were more sensitive to gemcitabine chemotherapy than the RRM1^−^ERCC1^+^ subpopulation cells (p < 0.05). Each experiment was repeated three times.

RT-qPCR and Western blot analysis showed that the mRNA and protein levels of HER2 in the BIU-87 cell line were higher than those in the CCC-HB-2 and KK47 cell line, whereas the mRNA and protein levels of RRM1 were no significant different in these cells (Figure 4A). To further investigate the sensitivity of cells with different expression levels of TOP2A, HER2, RRM1 and ERCC1 to different drugs, we isolated four subpopulations of cells (TOP2A^+^ HER2^−^cells, TOP2A^+^ HER2^+^cells, TOP2A^−^ HER2^+^cells and TOP2A^−^HER2^−^cells) from BIU-87 cells by using FACS(Figure 4B). The separated TOP2A^+^ HER2^−^cells were more sensitive to pirarubicin than TOP2A^+^HER2^+^ cells (p ≤ 0.05, Figure 4C). The HER2^−^cells had increased sensitivity to pirarubicin. Additionally, RRM1^+^ERCC1^−^cells, RRM1^+^ERCC1^+^cells, RRM1^−^ERCC1^+^cells and RRM1^−^ERCC1^-^ cells were sorted from the BIU-87 cell line (Figure 4D). The RRM1^−^ERCC1^-^ cells were more sensitive to gemcitabine chemotherapy than RRM1^−^ERCC1^+^ cells (p ≤ 0.05, Figure 4E). These results indicate that HER2 or ERCC1 expression may influence the effect of TOP2A or RRM1 on cancer cell responses to inhibitors.

## 4 Discussion

TOP2A and RRM1 are abnormally expressed in different types of cancers and are associated with cancer development [20, 21]. Several studies have reported correlations between TOP2A expression levels and prognosis in some cancer patients and indicated that TOP2A expression is a prognostic factor of cancer [22, 23]. In addition, the sensitivity or resistance of a malignant cell to several anti-tumour drugs known as “topoII poisons” depends on the cellular expression level of topoII. In bladder cancer, studies indicate that the expression of TOP2A in tumour cells is closely related to the efficacy of intravesical instillation of anthracyclines [24]. Anthracyclines, such as TOP2A inhibitors, are widely used in the clinic- and are highly specific and selective [25, 26]. Anthracyclines form stable complexes by the covalent binding of TOP2A with DNA strands, resulting in DNA breakage and chromosomal aberration, and thus, exhibiting anticancer effects [5]. In the present study, the cell toxicity experiments showed that bladder cancer cells with high expression of TOP2A significantly inhibited cancer growth compared with cells with low expression. Moreover, the curative effect in patients with high TOP2A expression was better than that in patients with low TOP2A expression after the intravesical instillation of pirarubicin. Pirarubicin IVT may have a better effect in patients with high TOP2A expression. However, not all tumours with high TOP2A expression are sensitive to pirarubicin treatment. In tumours with high TOP2A expression, those with concurrent HER2 overexpression showed a higher recurrence rate, and the IC50 in the TOP2A^+^ HER2^+^ cells was higher than that in the TOP2A^+^ HER2^-^ cells. Both HER2 and TOP2A are located in 17q12-22. Many studies have also found that the amplification and overexpression of HER2 in bladder cancer are closely related to the tumour grade and stage and significantly correlated with poor disease-specific survival. The configuration of 17q21 amplifiers in cancer cells may influence the effect of HER2 on TOP2A inhibitors, thereby affecting the efficacy of these chemotherapeutic drugs [14]. The mechanism by which HER2 interferes with TOP2A still needs further investigation.

In terms of drug sensitivity, in contrast to TOP2A, a low expression of RRM1 was associated with a high rate of response to gemcitabine-containing regimens. Gemcitabine is an example of a pyrimidine drug. After entering the body, the phosphorylation products of gemcitabine completely inhibit RR activity, resulting in a low or absent formation of dNTPs, which prevents DNA synthesis and induces apoptosis, thereby preventing the proliferation of cancer cells [27]. RRM1 is a necessary precursor for the synthesis of RNAs participating in a series of processes involved in RNA reduction to DNA and is the only enzyme that can catalyse RNA substitution to DNA; therefore, RRM1 can be used in DNA repair processes [28]. The low expression of RRM1 may limit the function of DNA repair, which reduces the ability of tumour cells to be more sensitive to chemotherapeutic drugs. Bergman et al. [29] established a non-small cell lung cancer model in vivo to study the causes of gemcitabine resistance. The results showed that RRM1 was highly expressed in resistant cells and that the RRM1 gene was a key target in gemcitabine resistance. Our studies show that the RRM1 expression level was correlated with cell sensitivity to gemcitabine. A low RRM1 expression was associated with a better response to gemcitabine. Previous studies revealed that patients with lower expression of RRM1 had a relatively long survival period after chemotherapy, indicating that the RRM1 gene is a major indicator of gemcitabine resistance [30]. In addition, a better response to gemcitabine was observed in the tumours with a low expression of both RRM1 and ERCC1. As an important factor involved in nucleotide excision repair, ERCC1 contributes to platinum resistance by repairing platinum-induced DNA damage in tumour cells [31]. Therefore, tumours with a low expression of both RRM1 and ERCC1 may have weaker DNA repair capacity and are more sensitive to chemotherapy. Above all, to achieve a better chemotherapy effect and prevent tumour recurrence, the TOP2A, RRM1, HER2 and ERCC1 mRNA or protein expression levels in NMIBC tissues could be detected after NMIBC. Gemcitabine IVT could be used in patients with TOP2A-positive and HER2-negative expression, while pirarubicin could be used in patients with RRM1 and ERCC1-negative expression, but it still need to be confirmed by further investigation based on larger number of basic and clinical experiments.

## 5 Conclusion

Tumour expression of TOP2A, RRM1, HER2 and ERCC1 may associated with the sensitivity of NMIBC to pirarubicin or gemcitabine treatment. Evaluating the different genes expression may provide help in chemotherapeutic drugs selection.

## Declarations

### Author contribution statement

Zhifei Liu: Conceived and designed the experiments; Performed the experiments; Analyzed and interpreted the data; Wrote the paper.

Liyong Xing: Performed the experiments.

Yanfeng Zhu: Analyzed and interpreted the data.

Peng Shi & Gang Deng: Contributed reagents, materials, analysis tools or data.

### Funding statement

This work was supported by Medical research project of Hebei Health Commition [NO.20171274].

### Data availability statement

No data was used for the research described in the article.

### Declaration of interests statement

The authors declare no conflict of interest.

### Additional information

No additional information is available for this paper.

## Figures and Tables

**supplement figure1A-GAPDH:**
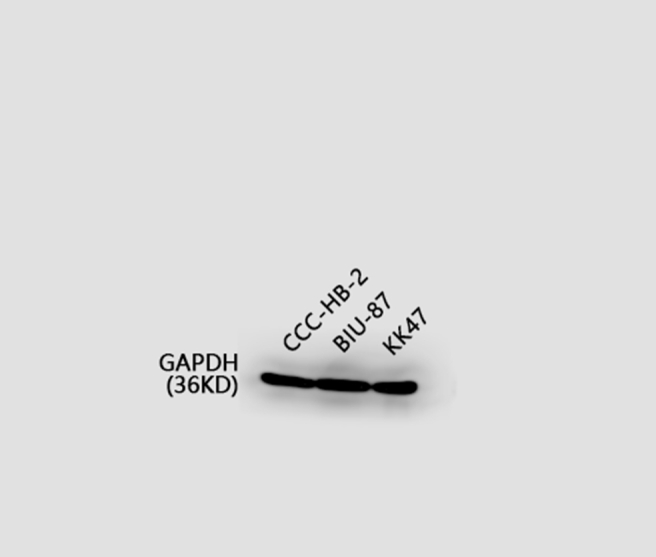


**supplement figure1A-RRM1:**
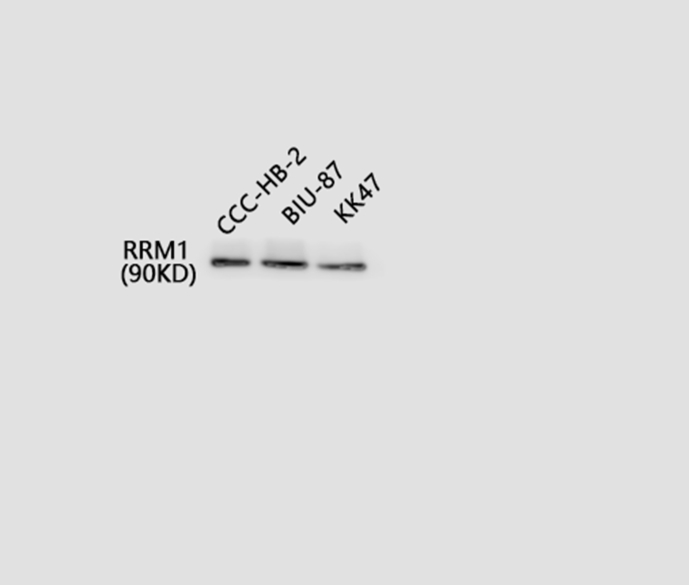


**supplement figure1A-TOP2A:**
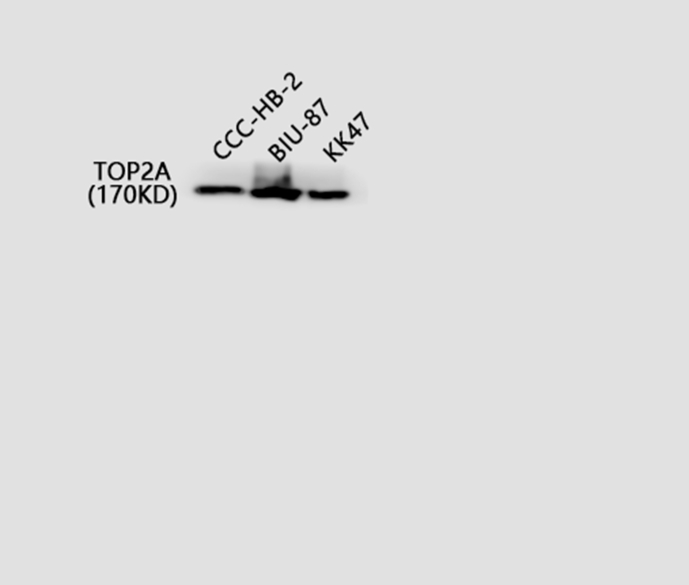


**supplement figure1B-GAPDH-TOP2A:**
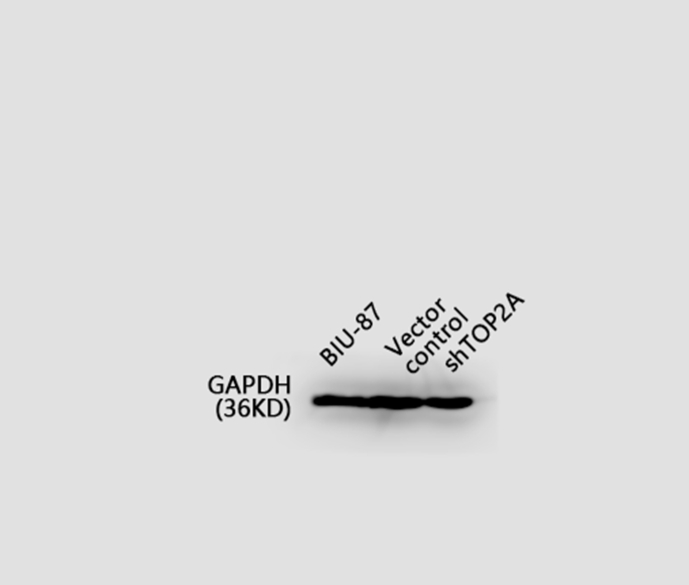


**supplement figure1B-RRM1:**
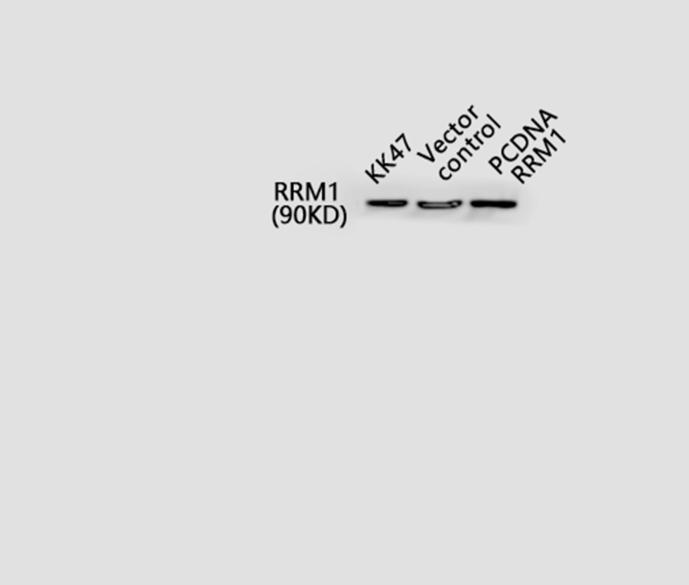


**supplement figure1B-TOP2A:**
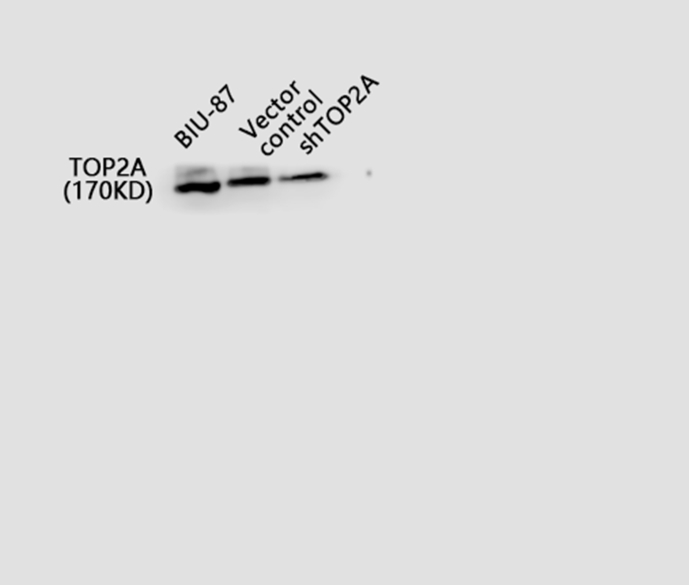


**supplement figure2A-ERCC1:**
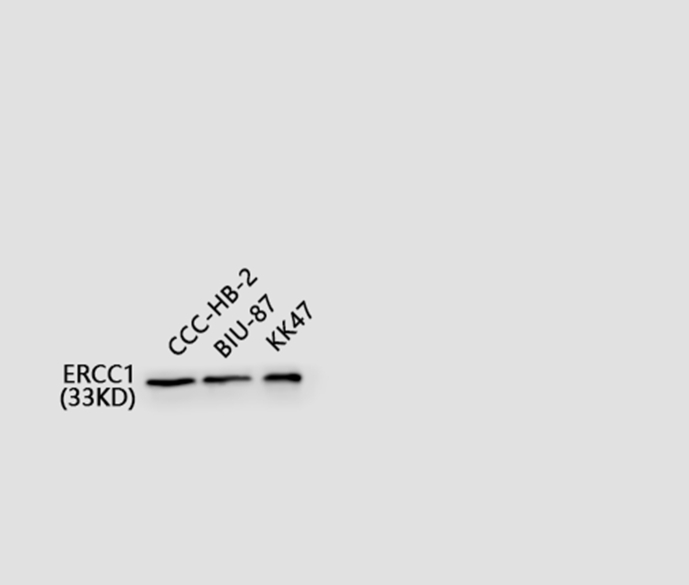


**Figure:**
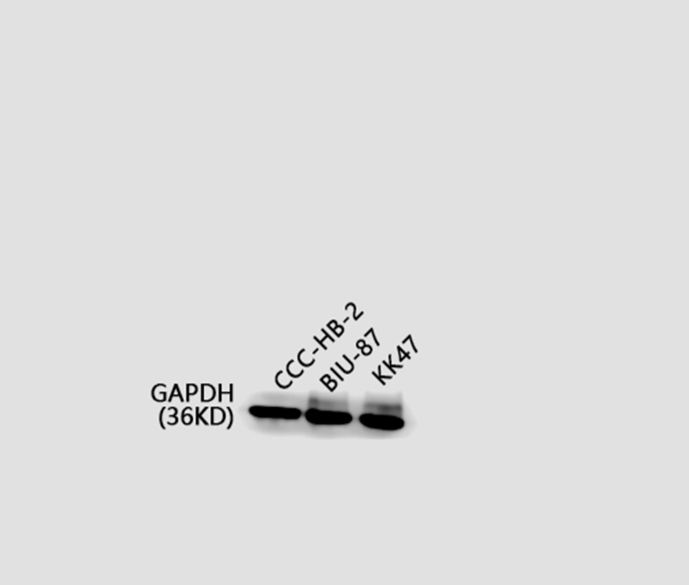
supplement figure2A-GAPDH

**supplement figure2A-HER2:**